# Deletion of the membrane complement inhibitor CD59a drives age and gender-dependent alterations to bone phenotype in mice

**DOI:** 10.1016/j.bone.2015.12.014

**Published:** 2016-03

**Authors:** Anja C. Bloom, Fraser L. Collins, Rob J. van't Hof, Elizabeth S. Ryan, Emma Jones, Timothy R. Hughes, B. Paul Morgan, Malin Erlandsson, Maria Bokarewa, Daniel Aeschlimann, Bronwen A.J. Evans, Anwen S. Williams

**Affiliations:** a Institute of Infection and Immunity, School of Medicine, Cardiff University, Cardiff, UK; b Bone Research Group, Institute of Ageing & Chronic Disease, University of Liverpool, Liverpool, UK; c Matrix Biology and Tissue Repair, Dental School, Cardiff University, Cardiff, UK; d Institute of Molecular and Experimental Medicine, School of Medicine, Cardiff University, Cardiff, UK; e Arthritis Research UK Centre for Biomechanics and Bioengineering, Cardiff University, Cardiff, UK; f Department of Rheumatology and Inflammation Research, Sahlgrenska University Hospital, University of Göteborg, Gothenburg, Sweden

**Keywords:** CD59a, Bone, Osteoclast, Ageing, Micro-CT

## Abstract

Degenerative joint diseases such as osteoarthritis are characterised by aberrant region-specific bone formation and abnormal bone mineral content. A recent study suggested a role for the complement membrane attack complex in experimental models of osteoarthritis. Since CD59a is the principal regulator of the membrane attack complex in mice, we evaluated the impact of CD59a gene deletion upon maintenance of bone architecture.

*In vivo* bone morphology analysis revealed that male CD59a-deficient mice have increased femur length and cortical bone volume, albeit with reduced bone mineral density. However, this phenomenon was not observed in female mice. Histomorphometric analysis of the trabecular bone showed increased rates of bone homeostasis, with both increased bone resorption and mineral apposition rate in CD59a-deficient male mice. When bone cells were studied in isolation, *in vitro* osteoclastogenesis was significantly increased in male CD59a-deficient mice, although osteoblast formation was not altered.

Our data reveal, for the first time, that CD59a is a regulator of bone growth and homeostasis. CD59a ablation in male mice results in longer and wider bones, but with less density, which is likely a major contributing factor for their susceptibility to osteoarthritis. These findings increase our understanding of the role of complement regulation in degenerative arthritis.

## Highlights

• Deficiency of CD59a altered bone remodelling in gender-specific manner.
• Higher intrinsic complement activity in male mice likely increased the impact of CD59a deficiency on bone.
• In male CD59a-deficient mice, femurs had increased length, cortical bone volume, osteoclast activity, and reduced bone mineral density.
• CD59a deficiency in bone marrow precursor cells specifically increases osteoclast but not osteoblast formation in vitro.
• CD59a is expressed by osteoclasts and osteoblasts and constitutes a potentially important regulator of bone growth and homeostasis in males.

## 1 Introduction

The balanced activity of bone-forming osteoblasts and bone-resorbing osteoclasts maintains bone homeostasis [1]. Mechano-signalling and systemic mediators activate resting bone surfaces, induce remodelling, and modulate the recruitment and maturation of osteoclast precursor cells [2], [3], [4], [5]. Osteoclastogenesis and consequential resorption of bone induces osteoblast maturation and the secretion of bone matrix and hydroxyapatite [6]. Osteoclast and osteoblast function is therefore coupled during bone remodelling. In 1991, Sato et al. discovered that osteoblast–osteoclast cross-talk is modulated by complement components [7]. The complement (C) system consists of activation pathways and the membrane attack complex (MAC). The MAC is formed from complex assembly of C5b, C6, C7, C8, and multiple copies of C9; once formed, it will create a membrane pore which can lyse target cells. This assembly is inhibited by CD59 expressed on almost all autologous cells [8], [9]. The complement system is a vital component of innate immunity with diverse roles; recent studies support its potential involvement in regulating bone homeostasis. C3-deficient bone marrow cells have dysfunctional osteoclast differentiation [10] whereas wild type (WT) bone marrow stimulation with C3a or C5a with IL-1β causes elevated osteoclast differentiation [11].

There is indirect evidence for the regulatory capacity of complement proteins in bone metabolism. This can be seen in human rheumatoid arthritis [12], and experimental models of arthritis such as antigen-induced arthritis [13], collagen-induced arthritis [14], and induced osteoarthritis (OA) [15]. In each case, the complement system is a potent trigger and amplifier of local and systemic inflammation that causes bone atrophy and aberrant bone apposition. Complement proteins are also expressed in a specific spatial arrangement in the growth plate during bone development [16]. Differential modulation of complement-related genes during osteoblast differentiation has also been described [17]. Furthermore, osteoblasts express the central complement proteins C3 and C5 and their corresponding receptors C3aR and C5aR [11]. Therefore, complement and bone homeostasis are intricately linked.

The physiological impact of MAC activation upon bone, osteoclast differentiation, and osteoblast maturation has been largely neglected. However, a recent study using murine experimental models of OA identified MAC as a potential regulator of cartilage degeneration and osteophyte formation [15]. In OA models, mice lacking the CD59a gene (CD59a^−/−^) exhibited exacerbated cartilage erosion and synovitis, whereas deficiency in C5 or C6 protected mice from cartilage loss and synovitis. Furthermore, C5 and C6 deficiency prevented, whereas CD59a deficiency augmented osteophyte formation [15].

Research into complement regulators has been greatly advanced in genetically engineered mouse models [15]. Wang et al. showed that CD59a^−/−^ mice can develop osteoarthritis spontaneously as evidenced by cartilage loss, hence creating a model highly comparable to human disease [15]. We set out to explore the impact of CD59a upon bone formation and age-related changes to bone structure in mice. Our data reveal, for the first time, that CD59a is a regulator of bone growth and homeostasis.

## 2 Materials and methods

### 2.1 Animals

Experiments were performed using WT and CD59a^−/−^ [18] mice (C57BL/6J). Animals (8, 20, and 50 weeks of age) were bred in-house and maintained in conventional housing. Procedures were executed in accordance with UK Home Office Project Licence PPL-30/2361. Animals were randomly allocated into study groups.

### 2.2 Assessment of bone morphology

Hind limbs were harvested and fixed in 70% methanol. Femoral length was determined from contact X-rays using the KODAK In vivo Imaging System FX Pro (Carestream Health). Femoral width was measured using a digital calliper. Structure of distal femoral bones was assessed by micro-CT (Skyscan-1072 X-Ray microtomograph). High-resolution scans with an isotropic voxel size of 10.8 μm were acquired (49.6 kV, 200.8 μA, 1 mm aluminium filter, 0.45° rotation angle). A 1 mm section of trabecular bone from the metaphysis was analysed, starting 1 mm below the reference point within the growth plate. Cortical bone architecture was evaluated 3 mm below the same reference point (1 mm section measured). The scans were reconstructed using NRecon and analysed using the CTAn software package (Bruker-Micro-CT). For calculation of the bone mineral density (BMD), calibration of the Skycan CT system was performed with known density calcium hydroxyapatite phantoms (0.25 and 0.75 g/cm^3^).

### 2.3 TRAP staining for osteoclasts

Fixed femora were embedded in methylmethacrylate [19]. Osteoclasts were identified on longitudinal sections (5 μm) of the distal femurs using tartrate-resistant acid phosphatase (TRAP) staining [20]. TRAP staining was photographed using a Aperio ScanScope CS slide scanner (Leica Biosystems) and analysed using Image J-based software [21].

### 2.4 Immunohistochemistry for osteopontin

Hind limbs were fixed in neutral buffered formalin, decalcified in 10% formic acid for 3 weeks at 4 °C, embedded in paraffin wax, and cut in 7 μm thick sections. Osteopontin (OPN) expression was visualised using an ImmPRESS Detection Kit (Vector Labs) as per manufacturer's instructions adding 1 μg/ml of mouse anti-OPN antibody (AKm2A1, Santa Cruz) or isotype control (MOPC-31C, BD). Sections were counterstained with haematoxylin. Non-overlapping areas at × 40 magnification were randomly selected for analysis using Image J-based macros [21].

### 2.5 Histomorphometric analysis of bone formation

Osteoid was observed in methylmethacrylate embedded sections after staining with von Kossa/van Gieson reagents [19]. Mice received two intra-peritoneal injections of calcein (10 mg/kg dissolved in 2% Na_2_HCO_3_ in PBS) 4 and 1 days before sacrifice. Calcein double labelling was quantified in methylmethacrylate embedded sections counterstained with Aniline blue (0.3 mg/ml, pH 7.5).

### 2.6 *In vitro* osteoclastogenesis assay

Femurs were harvested from mice and the proximal ends removed to collect bone marrow cells (BMC) by centrifugation [22]. BMC were resuspended in α-MEM containing 10% heat-inactivated FCS and 50 units/ml penicillin–streptomycin (growth medium). BMC (6.4 × 10^4^) were added to 6 mm glass coverslips in petri-dishes. Following 2 h incubation at 5% CO_2_ and 37 °C, non-adherent cells were removed by washing in fresh medium and coverslips were transferred into wells (24-well plate) with media containing 25 ng/ml macrophage colony-stimulating factor (M-CSF) with or without 2 ng/ml RANKL (both R&D Systems). Media was exchanged on day 3 and cells were fixed in acetone on day 7. TRAP staining was performed according to manufacturer's instructions (acid phosphatase kit, Sigma–Aldrich). Five fields of view on each glass coverslip at × 40 objective magnification were counted for total cells and TRAP-positive multinucleated cells (≥ 2 nuclei) [23].

### 2.7 Quantification of CXCL1/mKc by enzyme-linked immunosorbent assay

Murine keratinocyte-derived cytokine (mKc) levels were quantified from osteoclastogenesis assay supernatants following the manufacturer's protocol (R&D Systems).

### 2.8 *In vitro* osteoblast formation assay

BMC were harvested as described above and osteoprogenitors cultured in α-MEM containing 20% FCS, 50 units/ml penicillin–streptomycin (expansion medium). Once confluent, cells were gently scraped from the culture surface and re-seeded at 4 × 10^4^ cells/well in 12-well plates. After 24 h, medium was replaced with growth media supplemented with 10 mM β-glycerophosphate, 50 μg/ml ascorbic acid, and 10 nM dexamethasone (mineralisation medium). Osteoblasts were cultured for 14 days, changing media every 3–4 days. Alkaline phosphatase (ALP) activity was identified using SigmaFast 5-bromo-4-chloro-3-indolyl-phosphate (BCIP)/nitro blue tetrazolium (NBT) stain (Sigma–Aldrich). Alizarin red staining was used to visualise calcium phosphate deposition in the matrix. Stained plates were scanned and the percentage of the well covered by ALP-positive cells or mineral determined using Image J.

### 2.9 Statistical analysis

Analyses were performed with Graphpad Prism v5. A Student t-test was performed when comparing 2 groups. Two-way ANOVA with Bonferroni post-tests were utilised when assessing more than 2 groups with 2 independent variables, respectively.

## 3 Results

CD59a-deficient mice were shown to develop more severe arthritis [24]. Although lack of CD59a was studied extensively in disease models such as arthritis, involvement into homeostatic regulation is poorly defined. Therefore, we examined bone growth in naive CD59a-deficient mice.

### 3.1 Male CD59a-deficient mice have enhanced bone growth

**Fig. 1:**
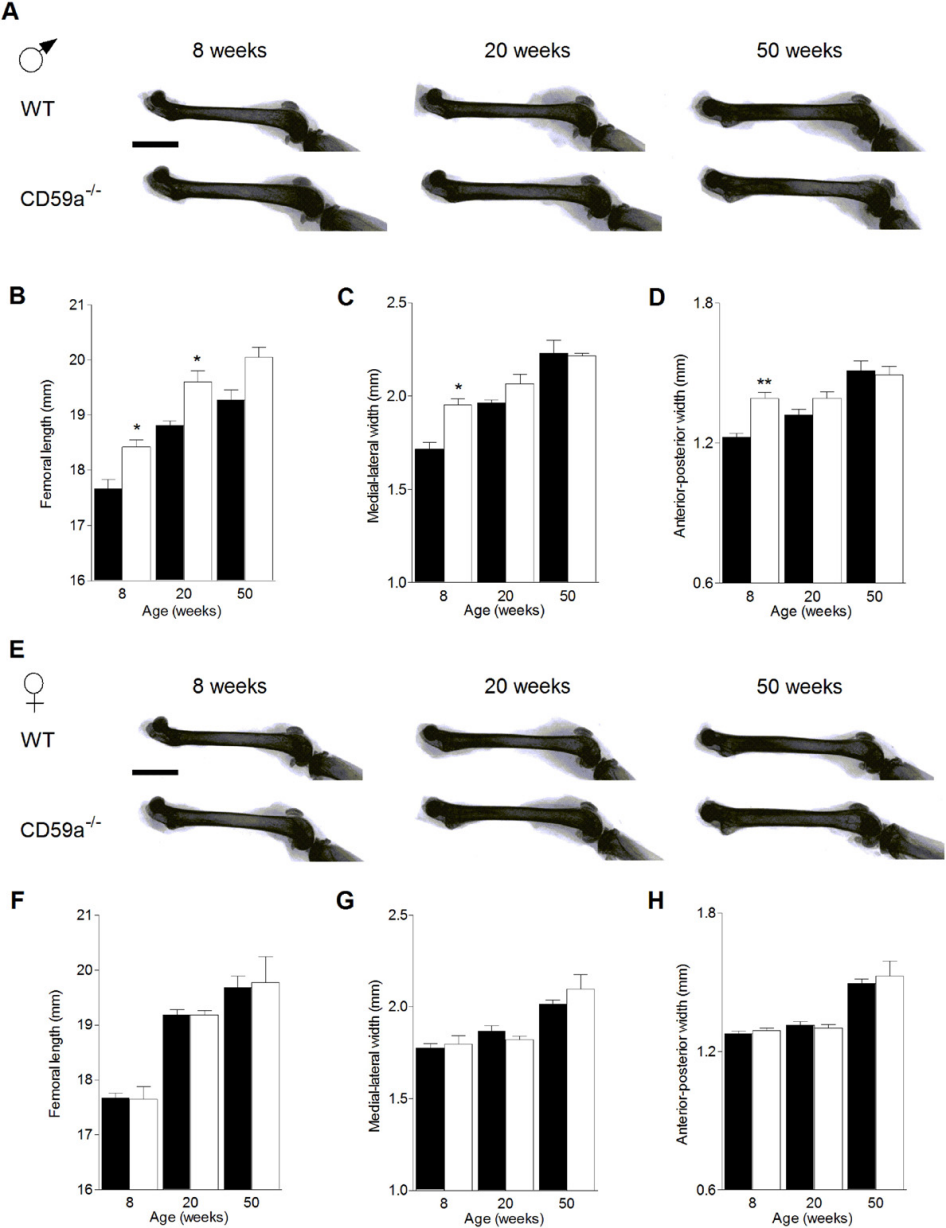
Bone growth is increased in male CD59a-deficient mice. Femurs were X-rayed and width measured using digital calliper. (A) Representative images of male 8–50-week-old WT and CD59a^−/−^ mouse femurs showing differences in bone size. Scale bar (black): 5 mm. (B) Femoral length in male WT and CD59a^−/−^ mice. (C) Medial–lateral femoral shaft width and (D) anterior–posterior femoral shaft width in male WT and CD59a^−/−^ mice. (E), (F), (G), and (H), respectively, show representative x-rays, femoral length, medial–lateral femoral shaft width, and anterior–posterior femoral shaft width in age-matched female mice. All values are mean ± SEM for six WT (black bars) and CD59a^−/−^ (white bars) mice per group. ^⁎^P < 0.01, ^⁎⁎^P < 0.001 versus WT of the same age and sex.

Bone morphology measurements revealed that femoral length increased with age in both male and female mice (Fig. 1). Significantly longer femurs due to CD59a deficiency were observed in male mice (CD59a^−/−^ versus WT) at 8 and 20 weeks of age (Fig. 1A to D). There was no difference between female WT and CD59a^−/−^ mice at any time point (Fig. 1E to H). Femoral width (measured in medial-lateral (Fig. 1C) and anterior–posterior direction (Fig. 1D)) was significantly increased in male CD59a^−/−^ mice during postnatal growth phase (8 weeks) but not after reaching maturity (20 weeks). There was no significant difference in body weight between the two strains; in agreement with published data [18].

### 3.2 Male CD59a-deficient bones have more bone volume which is less dense

**Fig. 2:**
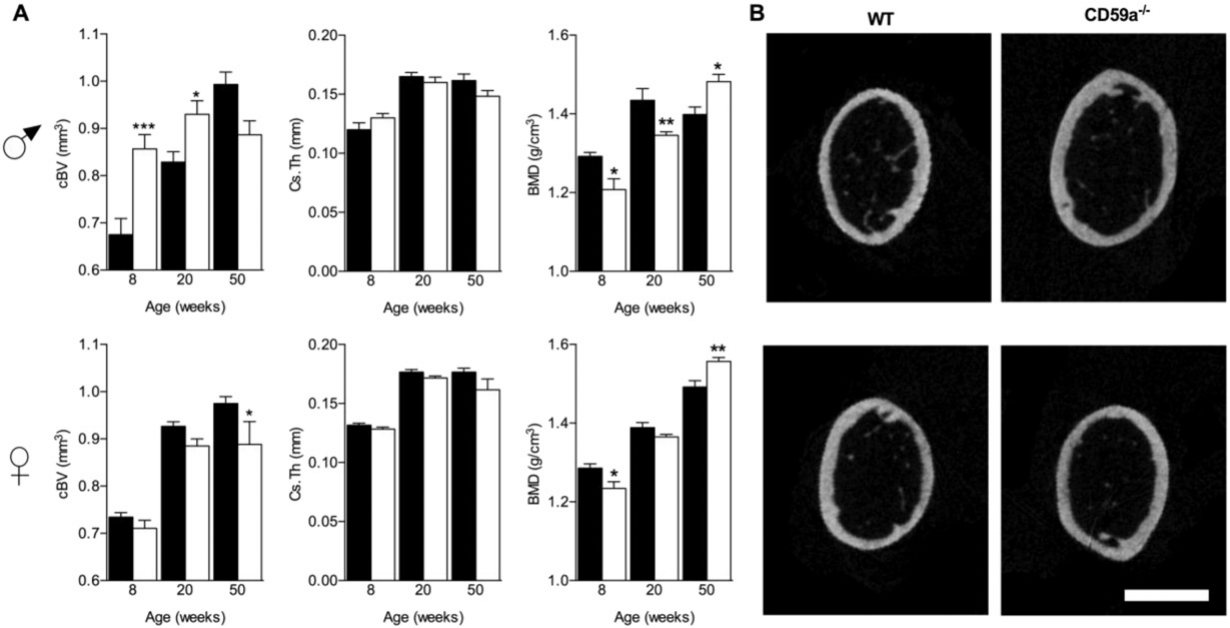
Altered cortical bone properties in young-adult CD59a-deficient male mice. Femurs were subjected to micro-CT and cortical bone of the shaft was investigated. A 1 mm section of cortical bone from the metaphysis was analysed; starting 3 mm below a reference point within the growth plate. Trabecular bone architecture (reported in Fig. 3) was measured in a section that started 1 mm below the same reference point. (A) Cortical bone volume (cBV), cross-sectional thickness (Cs.Th) and bone mineral density (BMD) of male and female 8–50-week-old WT (black bars) and CD59a^−/−^ (white bars) mice are shown. All values are mean ± SEM from a minimum of six mice per group. ^⁎^P < 0.05; ^⁎⁎⁎^P < 0.001 versus WT of the same sex. (B) Representative images at corresponding relative positions of 8-week-old WT and CD59a^−/−^ mouse femur cross-sections illustrating variations in bone diameter. Scale bar (white): 1 mm.

When studying bone architecture, cortical bone volume (cBV) was significantly increased in male and female WT mice over time; at 8 and 20 weeks, cBV was augmented in male CD59a^−/−^ mice (Fig. 2A and B). Analyses at 50 weeks of age showed small reduction in cBV (Fig. 2A, upper panel). In females, cBV was comparable at 8 and 20 weeks but decreased significantly at 50 weeks of age compared to WT femurs (Fig. 2A, lower panel). Tissue volume mirrored the increase in cBV observed in young adult CD59a^−/−^ male mice (significant percentage increase at 8 [20%], and 20 [13%] weeks; not shown). Cross-sectional thickness (Cs.Th) of cortical bone was not significantly altered in male or female mice (WT versus CD59a^−/−^). BMD increased steadily over time in CD59a^−/−^ (male and female) and WT (female only) mice. In WT male mice, BMD increased between the ages of 8 and 20 weeks and plateaued thereafter. BMD was significantly lower in male mice (CD59a^−/−^ versus WT) at 8 and 20-week-old and female mice (8 weeks only). In 50-week-old mice (male and female), BMD was significantly higher in CD59a^−/−^ compared to WT (Fig. 2A). Osteophyte formation was not observed but some irregularities at the subchondral bone occurred at 50 weeks of age (data not shown).

**Fig. 3:**
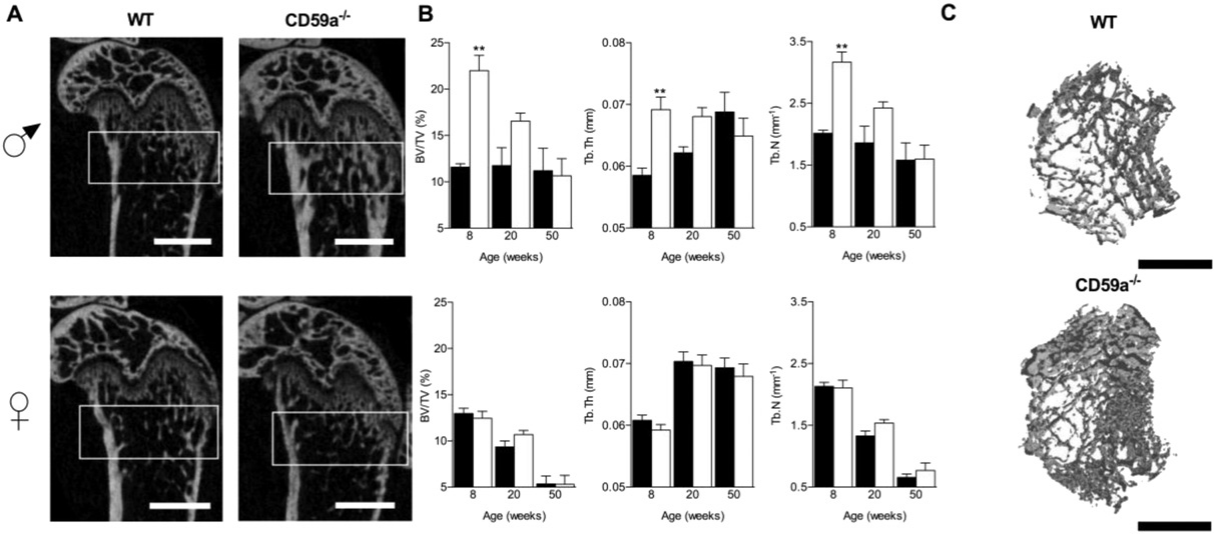
Structural changes in trabecular bone in male CD59a-deficient mice. The secondary spongiosa of femurs were analysed by micro-CT. A 1 mm section of trabecular bone from the metaphysis was analysed; starting 1 mm below a reference point within the growth plate. Cortical bone architecture (reported in Fig. 2) was measured in a section that started 3 mm below the same reference point. (A) Representative images of 8-week-old WT and CD59a^−/−^ mouse femurs, demonstrating marked increase in trabecular bone architecture compared to controls in male but not female CD59a^−/−^ mice (white boxes highlight region used for measurements). Scale bar (white), 1 mm. (B) Analysis of relative bone volume (BV/TV), trabecular thickness (Tb.Th), and trabecular number (Tb.N) is shown for 8, 20, and 50-week-old CD59a^−/−^ (white bars) and WT (black bars) mice. All values are mean ± SEM from a minimum of six mice per group. ^⁎⁎^P < 0.01 versus WT of the same sex. (C) Representative three-dimensional reconstructions from male 8-week-old WT and CD59a^−/−^ mice. Scale bar (black), 1 mm.

In trabecular bone, percentage bone volume (BV/TV) was significantly increased in 8-week-old male CD59a^−/−^ in comparison to age-matched WT mice (Fig. 3 A, B; upper plates) due to increased trabecular thickness (Tb.Th) and trabecular numbers (Tb.N; Fig. 3B and C). Differences were smaller at 20 weeks and at 50 weeks. Female CD59a^−/−^ mice did not show any difference in trabecular bone architecture compared to WT controls (Fig. 3 A, B; lower plates). As the bone phenotype was most prominent in young male CD59a^−/−^ mice, all remaining studies excluded female and 50-week-old mice.

### 3.3 CD59a-deficient mice show increased bone turnover

**Fig. 4:**
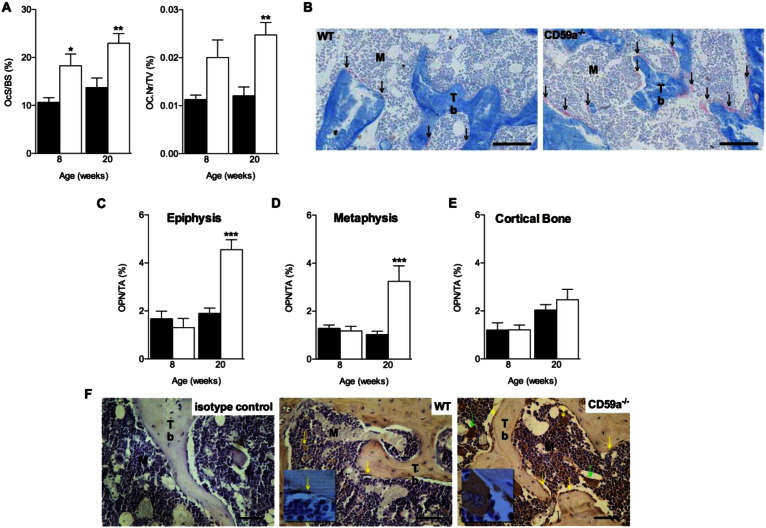
Elevated osteoclast activity and increased number of OPN-positive cells in trabecular bone in male CD59a-deficient mice. Femurs were stained with TRAP to identify osteoclasts. (A) Osteoclast surface per unit bone surface (OcS/BS) and osteoclast numbers per unit tissue area (OC.N/TA) were significantly increased in male CD59a^−/−^ (white bars) compared with WT (black bars) mice. All values are mean ± SEM from a minimum of six mice per group at 8 and 20 weeks of age. ^⁎^P < 0.05; ^⁎⁎^P < 0.01 versus WT. (B) Representative images of TRAP-positive osteoclasts (stained red and highlighted by arrow). M, marrow; Tb, trabecular bone. Scale bar (black), 100 μm. Femurs were stained for OPN by immunohistochemistry. Summary of analysis in (C) epiphysis, (D) metaphysis, and (E) cortical bone of male WT (black bars) and CD59a^−/−^ (white bars) mice at 8 and 20 weeks. All values are mean ± SEM from seven mice per group. ^⁎⁎^P < 0.01, ^⁎⁎⁎^P < 0.001 versus WT. (F) Representative images of isotype control and OPN staining (brown appearance and highlighted by yellow arrows) at 20 weeks. Megakaryocytes are shown by green arrows. Insets show higher magnification of staining. M, marrow; Tb, trabecular bone. Scale bar (black), 50 μm.

Bone histomorphometry showed increased osteoclast surface activity (osteoclast surface/bone surface; OcS/BS) in the secondary spongiosa of CD59a^−/−^ compared to WT mice at 8 and 20 weeks. Osteoclast number (OC.N/TA) was significantly increased at 20 weeks only (Fig. 4A and B).

Immunohistochemical staining of femurs for OPN revealed a significant 2.4 fold increase in CD59a^−/−^ versus WT mice at 20 weeks only (Fig. 4C and D). OPN was concentrated in cells that lined the bone (osteoblasts, osteoclasts, and bone lining cells) and megakaryocytes (Fig. 4F).

**Fig. 5:**
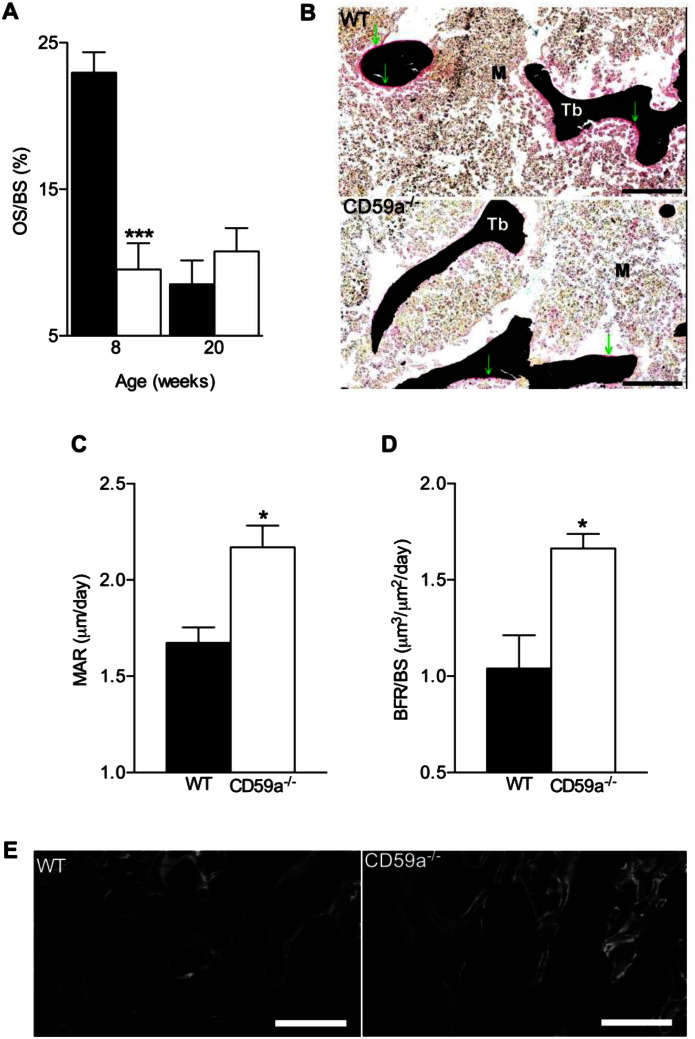
Reduced osteoid and increased bone formation in male CD59a-deficient mice at 8 weeks of age. Femurs were stained with von Kossa/van Gieson to reveal osteoids. (A) Histomorphometric investigation of femoral osteoid surface over total bone surface (OS/BS) of WT (black bars) and CD59a^−/−^ (white bars) male mice at 8 and 20 weeks. All values are mean ± SEM from a minimum of six mice per group at 8 and 20 weeks of age. ^⁎⁎⁎^P < 0.001 versus WT. (B) Representative images of osteoid staining with van Gieson (pink appearance and highlighted by arrows) at 8 weeks. M, marrow; Tb, trabecular bone. Scale bar (black), 100 μm. (C) Mineral apposition rate (MAR) and (D) bone formation rate (BFR/BS) calculated from calcein double labelling are shown in WT (black bars) and CD59a^−/−^ (white bars) mice. Bone formation rate is the amount of mineralised bone formed per unit of time per unit of bone surface. All values are mean ± SEM from four mice per group at 8 weeks of age. ^⁎^P < 0.05 versus WT. (E) Representative images of calcein double labelling in WT and CD59a^−/−^ mice. Scale bar (white), 100 μm.

Osteoclasts and osteoblasts act in a dynamic equilibrium as part of the bone remodelling unit in which osteoblasts secrete bone matrix [25]. When osteoblast activity was analysed by measuring osteoid surface/bone surface (OS/BS), in male CD59a^−/−^ mice at 8 weeks of age, OS/BS ratio was two-fold lower than in WT secondary spongiosa (Figure 5A and B). By 20 weeks, the OS/BS in WT mice was significantly reduced compared to 8 weeks and was not significantly different from the OS/BS in CD59a^−/−^ mice, the latter stayed unchanged between the time points (Figure 5A). MAR and BFR/BS were determined *in vivo* from double labelling with calcein (Figure 5C–E); both were significantly increased (MAR: 30%; BFR/BS: 60%) in the secondary spongiosa of CD59a^−/−^ compared to WT mice at 8 weeks of age. The increased calcein double label width of CD59a^−/−^ mice is clearly visible in Figure 5E. No adverse effects were observed.

### 3.4 CD59a deficiency promotes osteoclastogenesis but not osteoblast formation from BMC *in vitro*

**Fig. 6:**
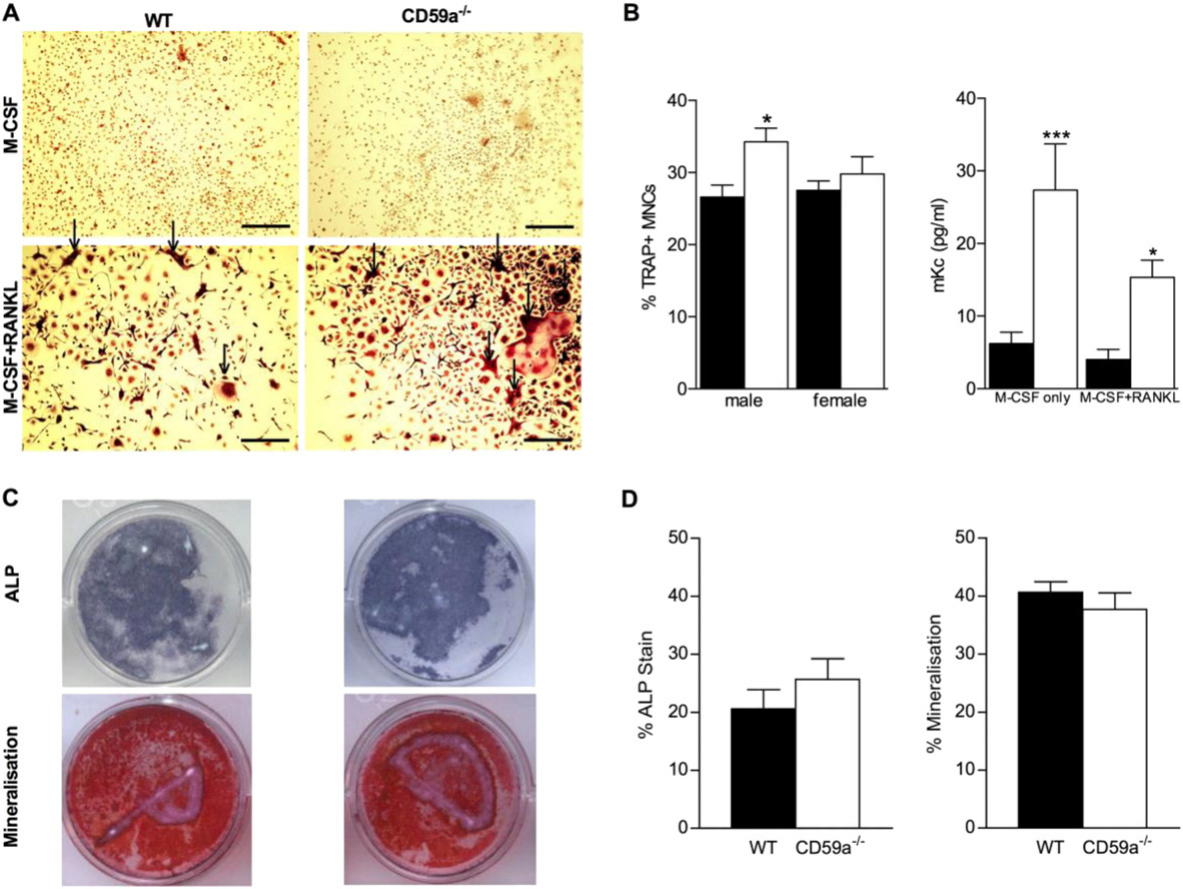
Increased osteoclastogenesis in CD59a-deficient mice in vitro. Osteoclast differentiation assays were conducted with RANKL-induced primary BMC from male and female WT and CD59a^−/−^ mice at 8–10 weeks of age. (A) Representative images of TRAP-negative control samples stimulated with M-CSF only and TRAP-positive cultures stimulated with M-CSF and RANKL (multinucleated cells [MNCs], arrow). Scale bar (black), 0.25 mm. (B) Quantification of TRAP-positive MNCs produced from RANKL-induced cultures. Culture supernatants of cells grown in M-CSF only and M-CSF and RANKL from male mice were harvested and mKc concentrations were measured by ELISA in WT (black bars) and CD59a^−/−^ (white bars) samples. (C) Osteoblasts were generated from enriched primary osteoprogenitor cells from male mice and, after 14 days in mineralisation medium, stained for ALP and Alizarin red. Representative images are shown. (D) ALP and Alizarin red coverage of wells was quantified. All values are mean ± SEM of triplicate cultures from at least five separate mouse bone marrows for each group at 8–10 weeks of age. ^⁎^P < 0.05; ^⁎⁎^P < 0.01; ^⁎⁎⁎^P < 0.001 versus WT cultures.

Initially, osteoclasts were generated in *ex vivo* cultures of BMC from 8–10-week-old male WT and CD59a^−/−^ mice. While M-CSF stimulation alone did not induce osteoclast formation, RANKL supplementation strongly promoted osteoclastogenesis, visualised as TRAP-positive multinucleated cells, in cultures from both WT and CD59a^−/−^ mice (Figure 6A). Percentage of TRAP-positive cells was significantly greater in BMC from male CD59a^−/−^ compared to WT mice (Figure 6B; 27%). Subsequent osteoclast differentiation assays conducted using BMC from age-matched female CD59a^−/−^ compared to WT mice revealed no alteration in TRAP-positive cell number. Culture supernatants from male BMC osteoclastogenesis assays were harvested and analysed for mKc (CXCL1), a marker for osteoclast precursor cell recruitment [26]. Levels of mKc were significantly increased in CD59a^−/−^ compared to WT culture supernatants in response to M-CSF. Co-administration of M-CSF and RANKL substantially reduced mKc levels compared against M-CSF alone. Nevertheless, levels of mKc remained significantly higher in CD59a^−/−^ compared to WT samples. To determine whether CD59a affects osteoblast formation *ex vivo*, cultures were generated from enriched BMC osteoprogenitor cells harvested from male mice (8–10-week-old). ALP staining and mineralisation were not significantly different between cultures derived from male WT and CD59a^−/−^ mice after 14 days in mineralisation medium (Figure 6C and D). Additional assays using BMC from female mice were not performed for ethical reasons, as there was no significant impact of CD59a ablation upon osteoblast differentiation in male mice.

## 4 Discussion

There are several ways in which cells can protect themselves from complement activity. In humans, CD59 is unique as it is the only complement regulator that controls MAC assembly by preventing lytic pore formation. In mice, CD59a performs these functions [27] and it is expressed in almost all tissues [9], [18]. CD59a gene deficiency exacerbates the severity of many mouse models of human diseases such as atherosclerosis, neuropathology, and importantly, for this study, arthritis [24], [28], [29]. In each case, the contribution of the terminal pathway of the complement system and MAC assembly to tissue injury is increased in the absence of CD59a. Here we tested the effects of CD59a deficiency on bone homeostasis to develop an understanding of how CD59a might influence normal bone and joint architecture. Our results indicate that normal bone homeostasis and structure is disrupted in the absence CD59a, which could explain the susceptibility of CD59a-deficient mice to develop both spontaneous and exacerbated arthritis [15], [24]. This indicates that complement regulator CD59a is required for maintenance of normal bone homeostatic functions.

The complement system is a central element in host defense, clearance of immune complexes, and tissue homeostasis. The less known regulatory roles of the complement system on bone metabolism and repair were reviewed recently [30]. Both clinical and experimental data clearly suggest a crucial function of complement in disorders affecting bone and in bone regeneration; however, the underlying molecular mechanisms remain unclear. We consider that the actions of CD59a on MAC formation and on bone cell activities are one and the same. The influence of MAC on a variety of cell types is well characterised [31], [32], [33]. Since MAC triggers a range of activation events in diverse cell types, the effects revealed here by CD59a deficiency on bone cells add to the list of non-lethal actions of MAC. The fact that the complement system in male mice raised on the C57BL/6J background is more active compared to females supports this contention [18], [34], [35]. The expected impact of heightened complement activation on a background of CD59a deficiency would be greater in males. Here, structural changes in bone were more profound in male mice supporting the hypothesis. There are several inflammatory joint diseases (e.g. OA, systemic lupus erythematosus, and rheumatoid arthritis) where clinical data revealed higher intrinsic complement activity and complement-associated pathology that may also affect bone [15], [36], [37].

Although femoral length increased with age in male and female WT and CD59a^−/−^ mice, femurs of male CD59a^−/−^ were longer (8 and 20 weeks) and thicker (8 weeks) compared to WT mice, despite little difference in overall body weight. No such difference was observed in female mice. Male CD59a^−/−^ mice have increased complement activity compared to females and consequently greater intravascular haemolysis and erythrocyte turnover [18]. However, heightened complement activity in male mice is not limited to CD59a deficiency. This is why male mice are frequently chosen for studying complement-mediated diseases in experimental models [24], [38]. In male mice, the impact of the increased complement activity is likely greater in the absence of CD59a than in WT mice where the effect of the terminal complement pathway is regulated [29]. There is also the differing nature and level of circulating sex hormones that define male and female mice at different ages. Oestrogen, for example, is a major player in bone homeostasis [39]; it can inhibit osteoclastogenesis and osteoclast activity. Furthermore, oestrogen acts on osteoblast precursor cells increasing or decreasing their expansion and maturation [39]. In diseases such as OA and osteoporosis, oestrogen may have a protective effect; women with low levels of estradiol were significantly more likely to develop radiographically defined OA than women with higher levels of estradiol [40]. Hence, the gender-specific bone phenotype reported here likely resulted from the combined protective effect of oestrogen in females and exacerbated impact of enhanced complement activity in male CD59a^−/−^ mice.

Alterations to femoral cortical bone architecture were observed in male CD59a^−/−^ mice. Specifically, cortical bone volume (cBV) was significantly increased (8 and 20 weeks) while bone mineral density (BMD) was reduced. This implies that osteoblast-dependent bone matrix apposition exceeds the rate of bone-matrix mineralisation, or that osteoclast-dependent resorption of mineralised bone could exceed the mineralisation rate. Both would result in a larger, albeit softer, bone mass. The altered bone remodelling in male CD59a^−/−^ mice is also apparent in the overall increase in: the number and thickness of bone trabeculae, the fraction of trabecular bone volume (TV) that is occupied by mineralised bone (BV), and mineral apposition rate (MAR) (8 and 20 weeks). The alterations in bone remodelling can be explained through the action of the MAC; the increased haemolytic activity and erythrocyte turnover seen in male CD59a^−/−^ mice could impact this directly. However, aside from its role in the generation of a lytic pore, MAC also drives cell activation and proliferation in several contexts, inducing pro-inflammatory cytokine production and Th1 cell polarisation as examples [41]. These responses may indirectly impact upon bone homeostasis by altering osteoclastogenesis, as reported in inflammation-induced activation of T-cells; which up-regulate RANKL, enhancing osteoclastogenesis and bone erosion [42].

To further understand the subtle influence of CD59a gene deletion upon bone remodelling, osteoblast and osteoclast activity was assessed *in situ* and differentiation assays were performed *ex vivo*. Whereas histological data showed up-regulation of osteoclast resorption surface and mineral apposition by osteoblasts confirming increased bone remodelling, osteoblast maturation assays *ex vivo* were not affected in male CD59a^−/−^ mice. However, male CD59a^−/−^ bone marrow cells produced significantly more osteoclasts than WT controls cultured under identical conditions (with RANKL and M-CSF), suggesting that osteoclasts are the main driver of the altered bone phenotype in CD59a^−/−^ mice or that mouse osteoblasts do not express CD59a and are normally unresponsive to activation by the terminal complement pathway. The membrane-bound regulatory protein CD59 is expressed in human osteoblasts and osteoclasts [11]; however, expression data in mouse have not been reported previously. Here we show for the first time that osteoclasts, bone tissue (femur), and calvarial osteoblasts maintained in monolayer culture or as a 3D construct to obtain an osteocyte phenotype express CD59a (see Supplementary Figure S2). Interestingly, the alterations in osteoclastogenesis were also gender specific *ex vivo*, mirroring *in vivo* findings. *Ex vivo* cultures are removed from protective effects of oestrogen, but the precursor cells may already be primed towards a specific functional fate. Aguila et al. identified gender-specific responses to IL-7 in *ex vivo* cultured bone marrow cells. The authors saw reduced myeloid precursors with osteoclastic developmental potential in females, but not in males. The authors also suggest that this could be due to hormonal modulation of hematopoiesis [43]. Nadaka et al. described that murine haematopoietic stem cells divide more frequently in females and is dependent on oestrogen produced in the ovaries [44]. Therefore, it is likely that oestrogen or other sex hormones influence haematopoietic precursor cells in CD59a-deficient mice, but this remains to be investigated. All *ex vivo* experiments were conducted in the absence of exogenous complement (heat-treated serum was used throughout) suggesting either that 1) there is an intrinsic difference between the CD59a^−/−^ versus WT bone marrow cell responses in culture that is not linked to complement activation or MAC assembly, or 2) the cells produce complement proteins in culture. Bone homeostasis relies upon cross-talk between mesenchymal-lineage bone-forming osteoblast and hematopoietic-lineage bone-resorbing osteoclasts; our data suggest that osteoclasts orchestrate increased bone remodelling in male CD59a^−/−^ mice. A view that is also supported by the elevated plasma levels of carboxy-terminal collagen crosslinks (CTX-I) and osteocalcin (Supplementary Figure S1). Final conclusions may not be drawn until lineage-specific knockouts have been generated and their bone phenotype analysed.

Mechanistic clues may be derived from secreted factors where mKc and OPN stood out. CD59a^−/−^ osteoclasts *in vitro* had increased mKc production, possibly due to an increased activation state. The cytokine mKc is a potent neutrophil chemotractant [45], and its presence *in vivo* is indicative of tissue inflammation [46]. mKc and its receptor CXCR2 are also involved in bone development [26]. CXCR2 is highly expressed in haematopoietic cells [47] and osteoclast precursors; therefore, release of mKc could trigger autocrine fusion of osteoclast precursor cells to mature osteoclasts, potentially enhancing their function [48]. Moreover, mKc secretion by osteoblasts and the recent demonstration that this chemokine regulates bone marrow macrophage (osteoclast precursors) trafficking endorse a functional role for mKc in modulating bone physiology [26], [49]. Increased tissue expression of OPN was observed in bones from CD59a^−/−^ mice. Aberrant expression of OPN is associated with abnormal bone mineralisation [50] and enhanced osteoclast-dependent pit formation [51]. How CD59a targets these mediators remains to be investigated.

The possibility remains that expression of complement components such as C3, C5, and their respective receptors by primed osteoclasts and osteoblasts [7], [10], [11] also contribute to the bone phenotype observed in male CD59a^−/−^ mice. To formally test these ideas, a substantial body of additional research is needed. Nevertheless, our study provides evidence that complement regulator CD59a can control normal bone homeostasis and is needed for the formation of strong bone morphology. The absence of CD59a can exacerbate bone diseases such as arthritis [15], [24], and our study presents the possibility that these phenotypes are compounded by weaker homeostatic bone structures that exist before disease commencement. Increased understanding of complement control of homeostatic functions may allow us to tease apart mechanisms related to health or disease.
