# Supplementary material for: Deletion of the membrane complement inhibitor CD59a drives age and gender-dependent alterations to bone phenotype in mice
Source: Bone. 2016 Mar;84:253–61. doi: 10.1016/j.bone.2015.12.014 (PMC4764651; doi:10.1016/j.bone.2015.12.014)
Supplement: Supplementary file 1 — Supplementary material [file mmc1.docx]

**SUPPLEMENTARY MATERIAL**

**Supplementary Caption:**

**Supplementary Figure S1:** Increased plasma levels of CTX-I (A) and osteocalcin (B) in young male CD59a-deficient mice.

**Supplementary Figure S2:** CD59a is expressed by murine osteoclasts (OC) and osteoblasts (OB).


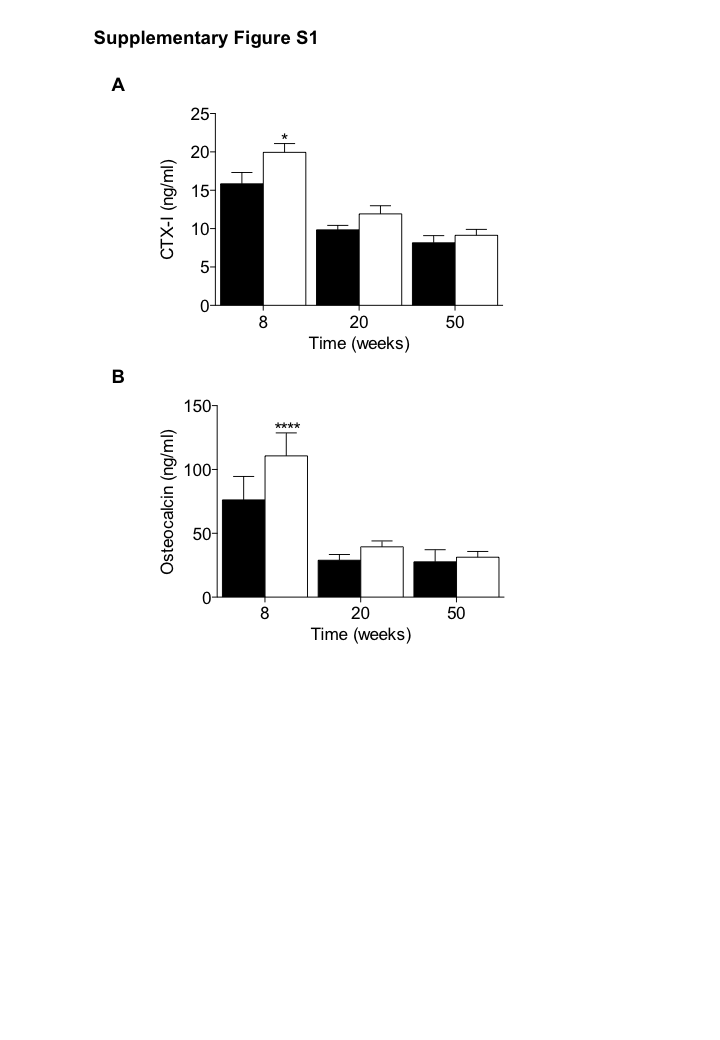


***Supplementary Figure S1.*** *Increased plasma levels of CTX-I and osteocalcin in young male CD59a-deficient mice. Blood was collected by cardiac puncture in heparin and plasma was obtained after centrifugation. Quantification of murine CTX-I (A) and osteocalcin (B) was performed by ELISA in WT (black bars) and CD59a^-/-^ (white bars) samples at 8-10, 20 and 50 weeks of age. ELISAs were performed according to manufacturer’s instructions (Immunodiagnostic Systems and Immunotopics Inc. respectively). All values are mean ± SEM from six separate mice per group. *P < 0.05; ****P < 0.0001 versus WT levels.*


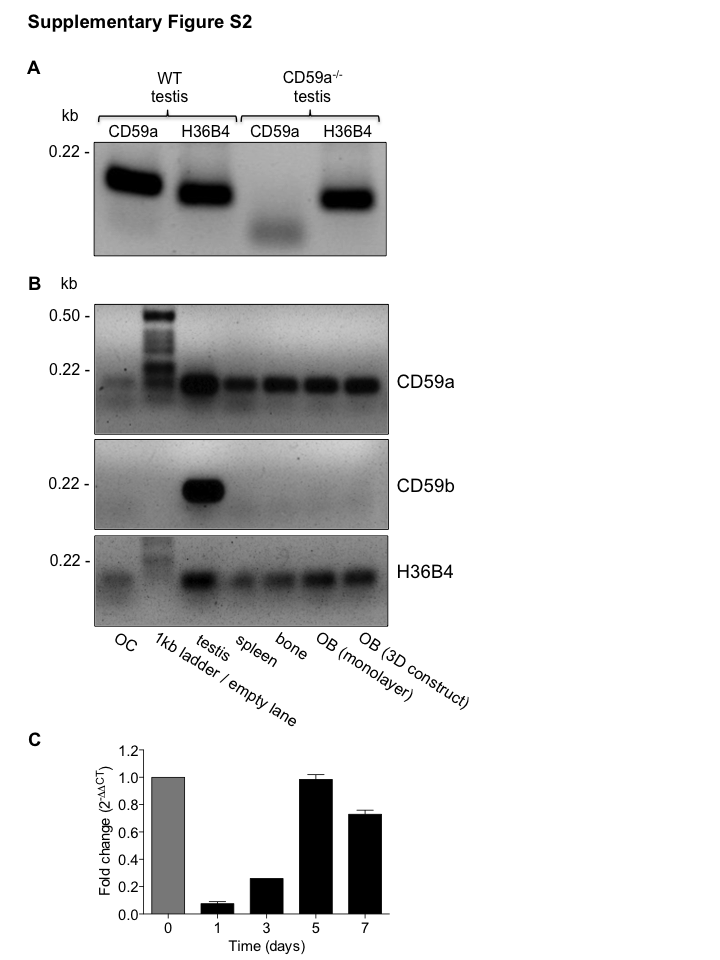


***Supplementary Figure S2****. CD59a is expressed by murine osteoclasts (OC) and osteoblasts (OB). (A) RT-PCR performed with poly(A^+^)-RNA obtained from testis of WT and CD59a^-/-^ mice demonstrates specificity of CD59a amplification. CD59a and CD59b are known to be abundantly expressed in germ cells in testis [*[*1*](#_ENREF_1)*]. PCR products were analysed in 1% agarose gels alongside a housekeeping gene (H36B4; [*[*2*](#_ENREF_2)*]) as a positive control. Whereas H36B4 was amplified in WT and CD59a^-/-^ mice, CD59a was only detected in WT samples. (B) Expression of CD59a in WT OC and OB was confirmed by RT-PCR. For this, poly(A^+^)-RNA was isolated from bone marrow cells differentiated with M-CSF and RANKL for 7 days to differentiate OC, bone tissue (femur), and calvarial OB maintained in monolayer culture or as a 3D construct to obtain an osteocyte phenotype, and analysed as in A. Unlike CD59a, CD59b expression was reported to be restricted to testicular germ cell elements [*[*1*](#_ENREF_1)*], hence testis and spleen were included in the analysis as a control. CD59b was only detected in the testis in contrast to CD59a which was expressed in all cells/tissues analysed, including OC and osteoblasts/osteocytes derived through endochondral and intramembraneous bone formation. (C) CD59a expression correlates with osteoclast development in differentiation cultures. To further analyse CD59a expression during osteoclastogenesis, quantitative PCR was performed for the 7-day time course of differentiation with M-CSF and RANKL. Expression was determined using poly(A^+^)-RNA and a TaqMan assay and is given as change relative to day 0 and normalized for housekeeping gene expression. As expected, levels of CD59a expression were high in bone marrow cell isolate (day 0; grey bar). Myeloid cells isolated by differential adhesion had low CD59a expression (day 1; black bar) but expression increased ~10-fold upon OC differentiation (day 5-7). Maximal induction between days 3 to 5 is consistent with what is seen for OC markers including cathepsin K [*[*3*](#_ENREF_3)*].*

**SUPPLEMENTARY REFERENCES**

[1] Harris CL, Hanna SM, Mizuno M, Holt DS, Marchbank KJ, Morgan BP. Characterization of the mouse analogues of CD59 using novel monoclonal antibodies: tissue distribution and functional comparison. Immunology 2003;109: 117-26.

[2] Wagener R, Kobbe B, Aszodi A, Aeschlimann D, Paulsson M. Characterization of the mouse matrilin-4 gene: a 5' antiparallel overlap with the gene encoding the transcription factor RBP-l. Genomics 2001;76: 89-98.

[3] Chen L, Wei XQ, Evans B, Jiang W, Aeschlimann D. IL-23 promotes osteoclast formation by up-regulation of receptor activator of NF-kappaB (RANK) expression in myeloid precursor cells. Eur J Immunol 2008;38: 2845-54.
